# [18F]ML-10 Imaging for Assessment of Apoptosis Response of Intracranial Tumor Early after Radiotherapy by PET/CT

**DOI:** 10.1155/2018/9365174

**Published:** 2018-06-10

**Authors:** Lu Sun, Kedi Zhou, Weijun Wang, Xiaojun Zhang, Zhongjian Ju, Baolin Qu, Zhizhong Zhang, Jinyuan Wang, Zhipei Ling, Xinguang Yu, Jinming Zhang, Longsheng Pan

**Affiliations:** ^1^Department of Neurosurgery, PLA General Hospital, 28 Fuxing Rd., Haidian District, Beijing, China; ^2^Department of Biomedical Engineering, College of Engineering, Peking University, 5 Yiheyuan Rd., Haidian District, Beijing, China; ^3^Department of Nuclear Medicine, PLA General Hospital, 28 Fuxing Rd., Haidian District, Beijing, China; ^4^Department of Radiation Oncology, PLA General Hospital, 28 Fuxing Rd., Haidian District, Beijing, China

## Abstract

[18F]ML-10 is a novel apoptosis radiotracer for positron emission tomography (PET). We assess the apoptosis response of intracranial tumor early after CyberKnife (CK) treatment by [18F]ML-10 PET imaging. 29 human subjects (30 lesions), diagnosed with intracranial tumors, underwent CK treatment at 14–24 Gy in 1–3 fractions, had [18F]ML-10 positron emission tomography/computed tomography (PET/CT) before (pre-CK) and 48 hours after (post-CK) CK treatment. Magnetic resonance imaging (MRI) scans were taken before and 8 weeks after CK treatment. Voxel-based analysis was used for the imaging analysis. Heterogeneous changes of apoptosis in tumors before and after treatment were observed on voxel-based analysis of PET images. A positive correlation was observed between the change in radioactivity (*X*) and subsequent tumor volume (*Y*) (*r*=0.862, *p* < 0.05), with a regression equation of *Y*=1.018*∗X* − 0.016. Malignant tumors tend to be more sensitive to CK treatment, but the treatment outcome is not affected by pre-CK apoptotic status of tumor cells; [18F]ML-10 PET imaging could be taken as an assessment 48 h after CK treatment.

## 1. Introduction

Intracranial and central nervous system (CNS) tumors are of high incidence in adolescents (0–19 years). In the U.S., the average age-adjusted incidence is annually 5.57 per 100,000 population from 2008 to 2012, and nearly 700,000 people live with intracranial and CNS tumors [1]. In the past decades, the overall diagnostic rate of intracranial tumor has been raised by the development of diagnostic imaging technologies, such as X-ray computed tomography (CT) and magnetic resonance imaging (MRI). Nowadays, the most common radical cancer treatments are surgery, radiotherapy, and chemotherapy. Also, radiotherapy is an important method for intracranial treatment that could either be a main regimen or adjuvant therapy after surgery for keeping cancer from recurrence. The evaluation of therapeutic response mainly relies on the change of tumor size assessed by CT and MRI images. For radiotherapy and chemotherapy, however, the anatomical volume change comes later than the rapid biological change on cellular level, resulting in a serious lag effect for the evaluation [2]. Indeed, the anatomical volume change of intracranial tumor is difficult to be visualized by CT or MRI till 4–8 weeks after whole brain radiotherapy [3, 4]. Particularly, for patients with malignant brain tumor, such as high-grade gliomas and metastatic tumors, and so on, the average survival time is short. With the lag effect of conventional assessments, the early response after radiotherapy is hard to be assessed which might result in a delay or loss of chance for alternative treatments [5–7]. Moreover, the tumor tissue and radionecrotic tissue could not be distinguished by CT and MRI images, which might cause ambiguous or even wrong judgement of therapeutic effect. Therefore, a method which could offer an early response assessment after radiotherapy for clinical management improvement is required.

Molecular imaging visualizes real-time biological process in cellular and molecular level by molecular probes. The development of molecular imaging makes early therapeutic assessment of radiotherapy feasible. As one of molecular imaging methods, apoptotic imaging could evaluate the early therapeutic response by providing dynamic apoptotic information after radiation, because radiotherapy cures cancer mainly by inducing apoptosis [8–10]. In the past decade, molecular imaging modalities such as PET/CT, MRI, magnetic resonance spectroscopy (MRS), B- Ultrasound (BUS) and diffuse optical tomography (DOT), and so on, have been developed and widely used. Among these modalities, only PET/CT could accurately provide quantitative information of apoptosis with proper apoptosis probe at molecular level.

Currently, three types of widely used apoptosis probes are proteins, nonspecific small molecules, and caspase activation. However, the limitations such as poor specificity, slow blood clearance, immunogenicity, and so on impede these probes' clinical applications. An ideal apoptosis probe for clinical practice should be specific for apoptotic cells, with rapid clearance, nontoxicity, and high stability [11–13]. [18F]-labeled 2-(5-fluoropentyl)-2-methyl malonic acid ([18F]ML-10), a low-molecular mass (molecular weight = 206) PET apoptotic tracer derived from Aposense family is the first clinically available apoptosis probe for in vivo imaging. Being investigated in multicenter preclinical and clinical trials, [18F]ML-10 shows high stability, safety, specificity, and rapid biodistribution [14, 15]. As a probe to visualize cell apoptosis in vivo, [18F]ML-10 selectively accumulates in apoptotic cells by recognizing alterations on the surface of apoptotic cells [16, 17], thus the apoptotic cells could be distinguished from normal cells. Additionally, [18F]ML-10 could be transported through cytoplasmic membrane in apoptotic cells, whereas there is no [18F]ML-10 membrane transportation in necrotic cells. Therefore, we are able to distinguish apoptosis from necrosis [18].

In this study, we investigated the performance of early response after CyberKnife (CK) stereotactic treatment on 29 patients with intracranial tumors through [18F]ML-10 PET/CT imaging with voxel-by-voxel analysis. Furthermore, the correlation between the result of this early assessment and subsequent anatomic change in tumor determined by MRI was also investigated for the further safety and effectiveness assessment.

## 2. Materials and Methods

### 2.1. Subjects

From January 2014 to December 2014, 29 human subjects (30 lesions) with intracranial tumors in our institution scheduled to undergo CK stereotactic radiotherapy were enrolled in the study. These patients were strictly selected in accordance with the integration and elimination standards, and their informed consents have been acquired.

The inclusion criteria were as follows: (1) the patient voluntarily subjects to this study, and the patient or legal representative signs the informed consents; (2) the patient is between 18 and 75 years old; (3) the patient has been diagnosed as intracranial tumor and meets the criteria for CK treatment; and (4) there are no abnormal findings on patients' routine blood, urine, and biochemical examination, electrocardiogram (ECG), and chest X-ray.

### 2.2. Informed Consent and Statement of Human Rights

Informed consent was obtained from all participants.

### 2.3. Imaging Protocol

#### 2.3.1. PET/CT Acquisition Protocol

PET/CT imaging was performed before and 48 hours after CK therapy with the AMIC Ray-Scan 64 PET/CT system (AMIC, Beijing, China), 90 minutes after [18F]ML-10 tracer injection with 0.12 mCi/kg b. w. The [18F]ML-10 tracer was produced at the Department of Nuclear Medicine at PLAGH PET Facility [19] with PET-MF-2V-IT-I fluorine multifunctional synthesis module (PETKJ, Beijing, China), and the radiochemical purity is of >98% as determined by HPLC.

PET images were acquired by three-dimensional brain mode, with 2.5 mm slice width and 512 × 512 reconstruction matrix. CT images were acquired with a 20 cm field of view (FOV), 75 cm diameter of gantry, 2.5 mm of slice width, 150 cm maximum positioning length, 175 cm axial moving range of patient bed, and 512 × 512 of reconstruction matrix.

#### 2.3.2. MRI Acquisition Protocol

MRI and CT scanning were used for location of tumor and anatomic assessment of the tumor response 2 to 4 months after radiotherapy. All MRI images were acquired on the 1.5 Tesla (1.5 T) MRI scanner (Siemens Espree, Siemen, Erlangen, Germany). Axial T1-weighted imaging (T1WI) was acquired, with slice width of 0.7 mm, repetition time (TR) = 1650, echo time (TE) = 3 ms; T2-weighted imaging (T2WI) was acquired, with slice width of 1.0 mm, TR = 5500, TE = 93 ms. 0.2 mmol/kg b.w. of gadolinium-diethylenetriamine pentaacetic acid (Gd-DTPA) was injected intravenously prior to 3D T1WI-enhanced imaging with above parameters.

#### 2.3.3. CT Acquisition Protocol

CT images for localization of tumor were acquired on Brilliance TM (Philips Healthcare, Amsterdam, Netherland), with 80 cm maximum field of view (FOV), 60 cm actual FOV, 85 cm diameter of gantry, 1.5 mm of slice thickness, 150 cm maximum positioning length, 190 cm axial range of patient bed, and 1024 × 1024 of maximum reconstruction matrix.

### 2.4. Radiotherapy Protocol

CyberKnife which enables stereotactic radiosurgery delivery applied in this study was manufactured by Accuray Inc., (Sunnyvale, CA, USA). A nonisocentric treatment plan was implemented through accelerator mounted on the robotic arm with continuous real-time image-guided technology.

The blood, urine, and biochemical examination, ECG, and chest X-ray were acquired before. After enrolment, patients underwent head CT and MRI scanning for location of tumor. For each patient, the acquired CT images was coregistered with MRI images by MIM software (version number: 6.5.4), which was imported into CyberKnife Robotic Radiosurgery System (Multiplan 4.0.2) for target and organs at risk delineation. With the information provided by the fused images, the target area of CK treatment was optimized, and the gross tumor volume (GTV) was recorded by radiologists and physicians. Patients underwent CK stereotactic radiosurgery with 6D⁃skull tracking, and the treatment scheme was with 14–24 Gy in 1–3 fractions depending on the tumor size and position.

### 2.5. Imaging Analysis

Voxel-based analysis on PET/CT image was used in the apoptosis imaging visual analysis, which performs voxel-by-voxel subtraction of the change PET/CT before (pre-CK) and early after CK treatment (post-CK). The PET/CT data of pre-CK and post-CK, along with CT, MRI, and GTV data, were delivered to the MIM image processing software after acquisition. The CT data with GTV information were registered with data from PET/CT imaging. With the imported GTV information as reference, region of interest (ROI) was plotted in MIM software. Each voxel value in PET/CT images was represented by radioactivity (Bq/ml) or standardized uptake value (SUV). The images of MRI, CT, and PET/CT were registered, and the voxel size and slice width from different imaging modalities were normalized. The values of radioactivity for each voxel in the ROI were collected separately on the baseline PET scan and the follow-up. The percentage change in each voxel was calculated. Voxel-based subtraction PET images were acquired using MIM software for visual analysis to observe the changes of cell apoptosis before and after CK treatment.

### 2.6. Statistical Analysis

For statistical analysis, two-tailed paired Student's *t*-tests were applied with SPSS 19.0 to analyze the change in radioactivity before and after treatment. Correlation between change ratio of radioactivity and lesion volume was analyzed with linear regression analysis. Also, *p* < 0.05 was considered as statistically significant.

## 3. Results and Discussion

### 3.1. Subjects and Lesions Information

In this trial, 33 patients aged from 22 to 69 years old, with 34 lesions, were included. Twenty-nine patients with 30 lesions completed the trial. Among the 30 lesions, there are 12 metastatic neoplasms (in 11 subjects), 6 meningiomas, 3 cavernous hemangiomas, 3 germ cell tumors, 2 hemangiopericytomas, 2 adenoid cystic carcinomas, 1 chordoma, and 1 hypophysoma. The 29 patients who completed the trial were with no radiation-related complication.

### 3.2. Visualization and Analysis of Apoptotic Imaging of Intracranial Tumor

**Figure 1:**
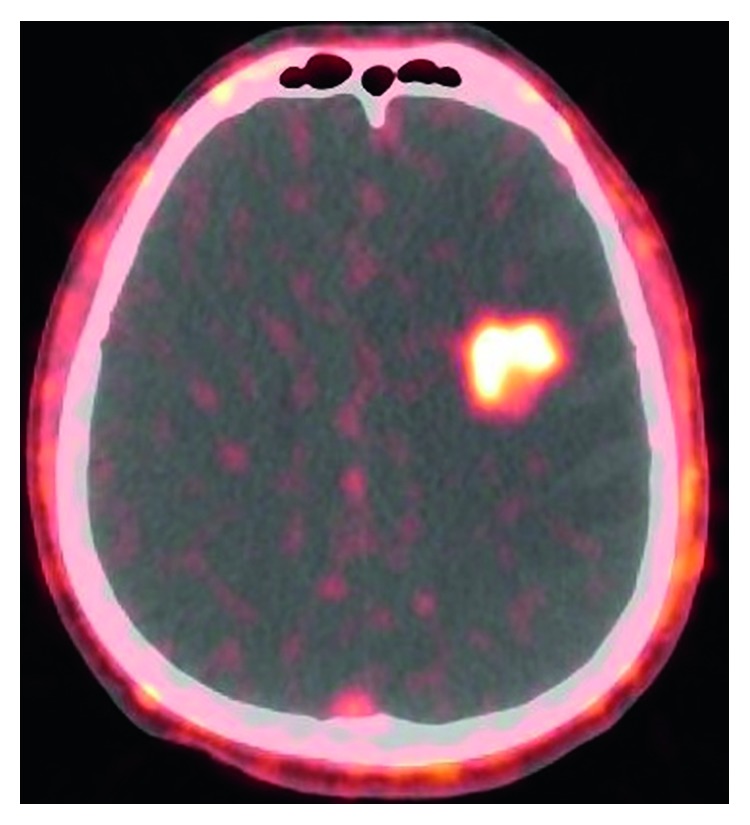
Representative PET/CT [18F]ML-10 image of a male patient, 48 years old, diagnosed with kidney cancer brain metastases, pre-CK.

The result of [18F]ML-10 PET/CT apoptosis imaging (Figure 1) shows a high PET signal from spontaneous apoptosis in the lesion area, while the signal in surrounding normal brain tissue is relatively low. Thus, the location and extent of the tumor could be identified in PET/CT images.

**Figure 2:**
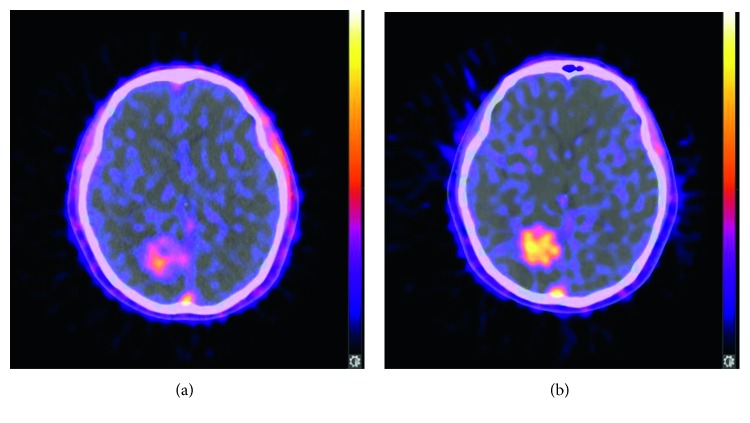
Pre-CK PET/CT image (a) and post-CK PET/CT image (b) of a female patient, 61 years old, diagnosed with lung cancer brain metastases, showing an obvious and uniform increase of [18F]ML-10 uptake.

**Figure 3:**
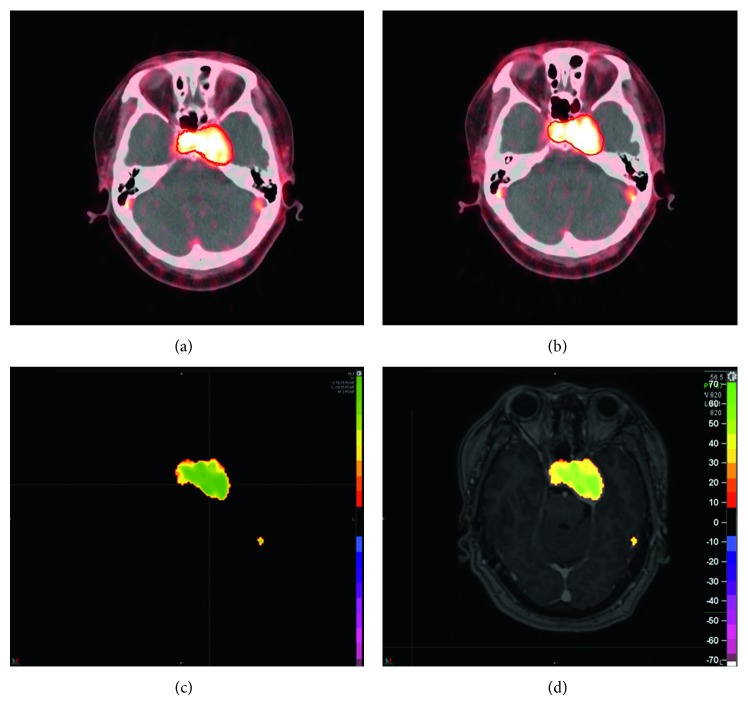
Pre-CK PET/CT image (a), post-CK PET/CT image (b), subtraction (c) of PET image, and fused PET/MRI images (d) of a female patient, 54 years old, diagnosed with cavernous hemangioma in cavernous sinus. Red line indicates the contour of tumor. The color bar shown in (d) corresponds to radioactivity changing ratio.

The change in [18F]ML-10 uptake between pre-CK (Figure 2(a)) and post-CK (Figure 2(b)) is analyzed by a voxel-wise method. As shown in Figures 2(a) and 2(b), the post-CK uptake of the ML-10 tracer is clearly higher than pre-CK uptake. In other cases, however, there is obscure difference of [18F]ML-10 uptake between pre-CK (Figure 3(a)) and post-CK (Figure 3(b)). As clear change in [18F]ML-10 uptake cannot be visualized by the PET/CT images, the subtraction of “post-CK”−“pre-CK” (Figure 3(c)) is processed by MIM software, with a reference of GTV information as the red contour of tumor. With this subtraction analysis shown in Figure 3(c), a greater change in the [18F]ML-10 was found in the central area of the tumor rather than the edge, suggesting that there are more apoptotic cells at the center while less apoptotic cells at the edge after CK treatment. In this case, an intuitive and clear change in the apoptotic tumor cells could be visualized by the change in [18F]ML-10 uptake with subtraction of PET images. Moreover, the area with high PET signal corresponded well with the lesions determined by the GTV and MRI fused image (Figure 3(d)).

**Figure 4:**
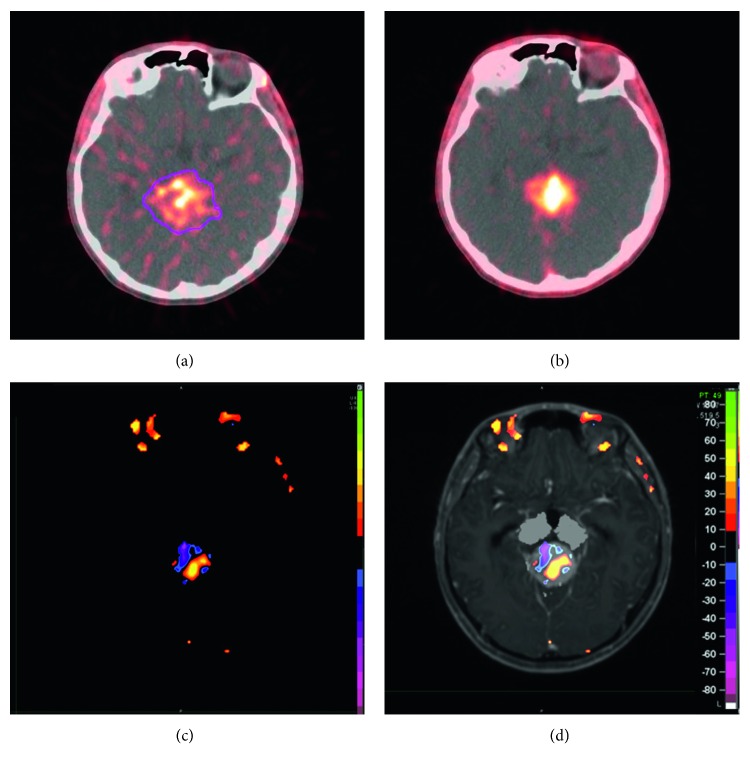
Pre-CK PET/CT image (a), post-CK PET/CT image (b), subtraction (c) of PET image, and fused PET/MRI images (d) of a male patient, 21 years old, diagnosed with germ cell tumor. Color bar shown in (d) corresponds to radioactivity changing ratio. The change in tracer uptake from pre-CK (a) to post-CK (b) is visualized by the subtraction of PET images (c) and PET/MRI images (d).

The analysis based on subtraction enables the judgement of whether there is more or less apoptosis happening in ROI after CK treatment. However, the heterogeneous and two-way overall changes of the tracer uptake cannot be revealed by subtraction analysis. As shown in Figure 4, some portion of the tumor becomes more apoptotic, while the other becomes less apoptotic, and also there are portions that remain unchanged. Therefore, quantitative analysis of the voxel-based subtraction is needed to further investigate cases with heterogeneous and two-way changes.

### 3.3. Quantitative Analysis for Accurate Subtraction

With the registration and fusion of pre-CK and post-CK PET images, the change in [18F]ML-10 uptake in radioactivity after CK treatment of each voxel in ROI could be extracted and sorted into one of the following three categories: (1) voxels with increased [18F]ML-10 uptake, defined as a positive change in radioactivity of more than 12.5% from pre-CK, representing cells in early apoptosis; (2) voxels with decreased [18F]ML-10 uptake, defined as a negative change in radioactivity of more than 12.5% from pre-CK, representing vascular occlusion and/or clearance of apoptotic cells; and (3) voxels without change in [18F]ML-10 uptake, defined as a change in radioactivity of less than 12.5%, wherein, the threshold of 12.5% is set in accordance with [2, 20].

**Figure 5:**
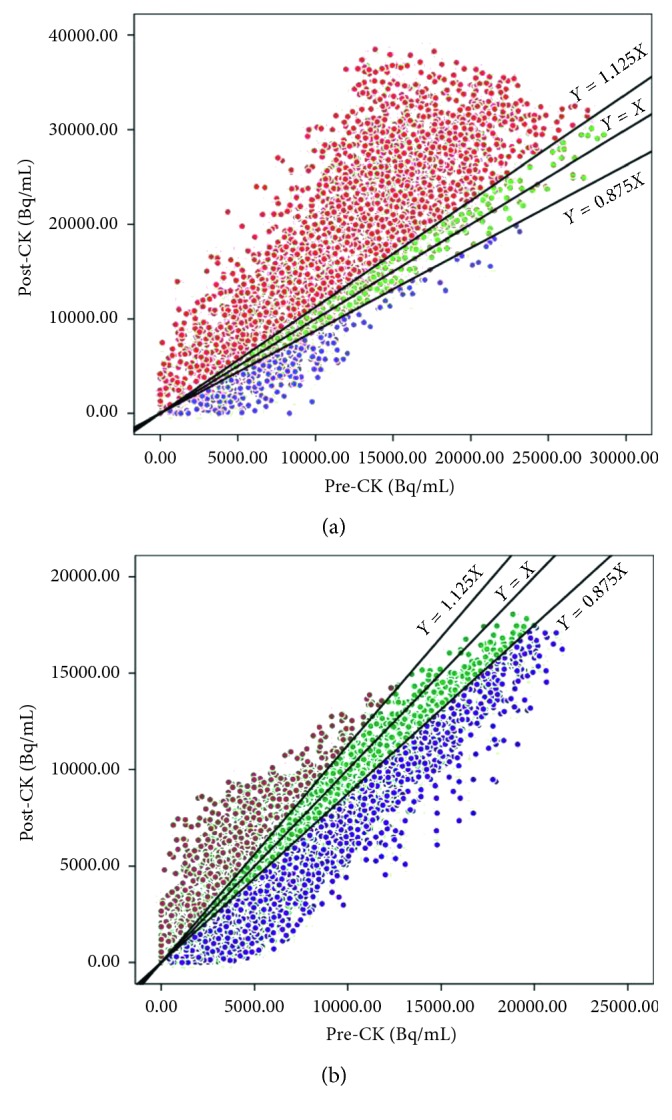
Voxel-based subtraction scatterplots showing the effect of CK treatment in a subject with positive apoptotic response (a) and a subject without significantly apoptotic response (b). The *X*-axes represent the pre-CK radioactivity while the *Y*-axes represent post-CK radioactivity. Voxels with increased signal are shown in red, representing increased apoptotic activities. Voxels with decreased signal are shown in blue, and voxels with unchanged (change less than ±12.5%) signal are shown in green.

As the change in apoptosis could not be accurately quantified by average change in radioactivity due to the heterogeneity of tumor tissue, the signal change in each voxel in ROI is plotted into a scatter graph (Figure 5), with pre-CK's radioactivity on the *X*-axis and the post-CK's radioactivity on the *Y*-axis. Voxels with increased signal are shown in red, representing increased apoptotic activities, voxels with decreased signal are shown in blue, while voxels with unchanged (change less than ±12.5%) signal are shown in green. The response of radiotherapy could be classified into different types, by the comparison of different subjects' signal changing pattern depicted in scatterplots (Figures 5(a) and 5(b)). As shown in Figure 5(a), the number of voxels with increased apoptosis indicated by red spots overwhelms that of decreased and unchanged signal, showing that positive apoptosis has been increased in the tumor area by SRS, thus suggests an effective radiotherapy. However, for the other case shown in Figure 5(b), no significant change in apoptotic signal could be defined as an increase or decrease by the scatterplot result. Therefore, the individual response early after radiotherapy could be revealed by quantitative scatterplot of apoptotic change, especially for heterogeneous change. Moreover, Moffat et al. assessed the effectiveness by correlating the early apoptotic change and the subsequent change in tumor volume [20].

**Figure 6:**
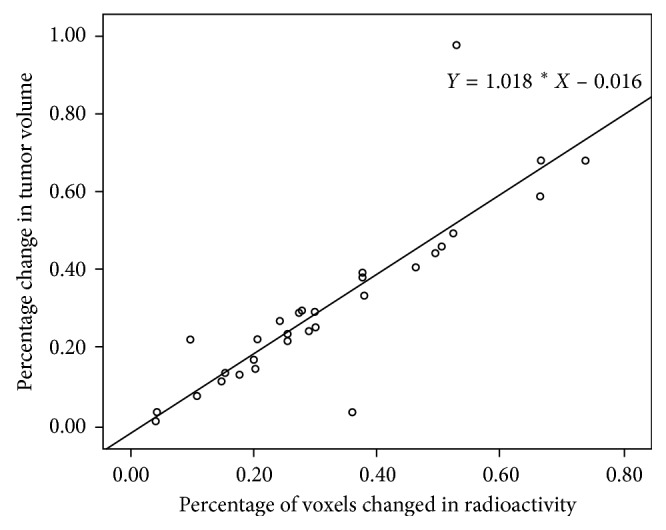
Correlation between the change in [18F]ML-10 uptake (*X*) and subsequent change in tumor volume (*Y*). The Pearson correlation coefficient is *R*=0.862, *p* < 0.05.

The change in tumor size for all 30 lesions from 29 subjects was obtained by MRI 2 to 4 months after CK treatment completion, and the mean percentage of volume change was 30.96% ± 21.73% (95% CI 22.85%–39.08%) reduced in tumor size. In the meanwhile, the mean change per voxel was 32.03% ± 18.40% (95% CI 25.16%–38.90%). As correlation analysis shown in Figure 6, a significant correlation was observed between the change in [18F]ML-10 uptake (*X*) and subsequent change in tumor volume (*Y*) with a Pearson correlation coefficient *R*=0.862, *p* < 0.05. The linear regression equation is *Y*=1.018*∗X* − 0.016. The *t*-test result of the regression coefficient is *t*=9.010, *p* < 0.05, and ANOVA result of the regression coefficient is *F*=81.175, *p* < 0.05.

### 3.4. Comparison of the Therapeutic Response in Different Cancer Types

**Figure 7:**
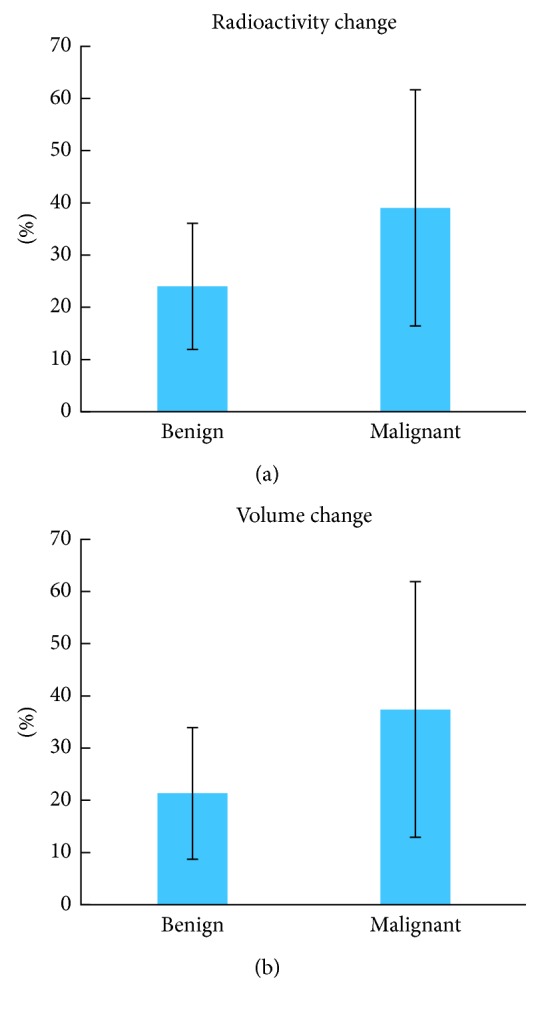
The comparison of therapeutic response in benign and malignant tumors. A significant difference of the radioactivity change (a) between malignant and benign tumor has been observed, *p*=0.0258. Furthermore, this difference is confirmed by subsequent volume change (b) with statistical significance, *p*=0.0262. The error bars represent standard deviation.

The correlation between apoptotic change and subsequent volume change has been revealed; it remains unclear that whether the apoptotic change was induced by treatment or by a high spontaneous apoptosis level in the tumor. To further investigate, the 30 lesions were divided into two subgroups by the level of uptake of [18F]ML-10 before treatment. Among all lesions included in this study, there are 16 lesions with a high pre-CK radioactivity value, while 14 lesions with lower radioactivity before CK treatment, with the threshold of 10,000. The comparison of therapeutic response between high pre-CK and low pre-CK groups was conducted. As shown in Figures 7(a) and 7(b), there is no significant difference in radioactivity change (*p*=0.5640) or subsequent volume change (*p*=0.7226) between these two groups after CK treatment. Therefore, the initial apoptosis level is not correlated with subsequent tumor shrinkage, and the observed change in tracer's uptake as measured is related to the CK treatment.

In accordance with 2016 World Health Organization's (WHO) classification of CNS tumors, 30 lesions in this study could be classified into 2 groups: 18 malignant and 12 benign tumors. The changes in radioactivity and subsequent tumor volume of each lesion have been analyzed, and the comparison in different cancer types is shown in Figure 7. As depicted in Figure 7(a), a significant difference of the radioactivity change between malignant and benign tumor has been observed, *p*=0.0258. Furthermore, this difference is confirmed by subsequent volume change with statistical significance (Figure 7(b)), *p*=0.0262. Therefore, malignant tumors could be considered to be more sensitive to CK treatment in comparison with benign tumors.

## 4. Discussion

Before the clinical use of [18F]ML-10 apoptosis imaging, [18F]FDG PET imaging has been used for the radiotherapy assessment in intracranial tumor in some studies [21, 22]. However, the increased [18F]FDG uptake caused by radiotherapy-related inflammation could lead to a false positive result in PET images. In addition, the high [18F]FDG uptake in normal tissue reduces the signal-to-noise ratio. Therefore, [18F]FDG imaging is not an ideal method for the assessment of intracranial tumor radiotherapy due to the limited accuracy. Lorberboym et al. [13] investigated apoptosis imaging of intracranial tumor by ^99m^Tc-Annexin-V SPECT imaging, which could achieve better accuracy. However, it was limited by the high molecular weight, slow blood clearance, immunogenicity, and poor specificity of ^99m^Tc-Annexin-V [23].

Early quantitative assessment of radiotherapy via noninvasive imaging is important to evaluate the treatment and then improve clinical management. In this study, [18F]ML-10 has been used to visualize the change of apoptosis in tumor area as an early assessment of CK treatment for intracranial tumor. With voxel-wise analysis as well as correlation analysis, the feasibility of the assessment method was demonstrated. The safety and efficacy of [18F]ML-10 have been investigated in preclinical studies [24] and multicenter clinical trials [15], suggesting good stability, safety, specificity, and rapid biodistribution.

The concept of apoptosis was first proposed by Kerr et al. [25]. A study on the difference between apoptotic cells and necrotic cells suggested that apoptosis is a programmed death process, the inhibition of which is highly related to the occurrence and development of tumor [26]. In addition, as B-cell lymphoma 2 (Bcl-2) gene is identified as a regulator of apoptosis, it is considered to be antiapoptotic, thus classified as an oncogene. Therefore, it is misunderstood that the apoptosis in tumor tissue is less active than normal tissue. In fact, more apoptosis was found in tumor tissue than normal tissue in most cases. In this study, a significantly higher [18F]ML-10 uptake in tumor cells was observed in PET/CT images.

Cancer is treated with radiotherapy mainly by apoptosis induction [8–10]. In the early stage of an effective treatment, complex pathophysiological changes occur in the tumor, including apoptosis onset in vascular endothelial cells, vascular occlusion, and consequent removal of necrotic cells, and so on. Especially, the tumor vascular occlusion and the removal of necrotic cells result in reduced tracer uptake visualized by apoptosis imaging, thus attenuates the overall apoptosis change in tumor tissue [2]. In this study, heterogeneous and two-way change of [18F]ML-10 uptake was observed in some subjects, as shown in Figure 4. Therefore, it may not be accurate to evaluate the efficacy of the treatment using the overall change of tracer uptake in ROI after treatment. Considering the spatial heterogeneity of tumor tissue, a voxel-based analysis method proposed by Moffat et al. [20] was used in this study. The PET signal of the whole tumor tissue was divided into voxels—the smallest unit of three-dimensional imaging. The change in radioactivity in each voxel was then calculated and classified into three categories (increase, decrease, and unchanged) with the threshold of 12.5%. For the quantitative analysis of the voxel-wise change, the voxels with both increased and decreased tracer uptake (red and blue points shown in Figure 5) were put together as tissue with apoptosis change induced by radiotherapy to take the heterogeneously internal changes from the tumor tissue into consideration. Positive correlation was observed between the change in [18F]ML-10 uptake (*X*) and the subsequent change in tumor volume (*Y*) with a linear regression equation: *Y*=1.018*∗X* − 0.016. Additionally, the difference of voxels with increased and decreased tracer uptake may provide potential reference to distinguish the apoptosis and necrosis in tumor tissue.

All 30 lesions have also been divided into two groups by pre-CK radioactivity, but no significant difference was found in radioactivity change or subsequent volume change, indicating that the therapeutic response of CK treatment is related to the CK treatment, rather than the apoptosis before CK treatment. Twenty-nine subjects with multiple types of intracranial tumor have been investigated in this study; 30 lesions in 29 patients were classified into malignant and benign, in accordance with 2016 WHO CNS tumors classification. A significantly different response in radioactivity, as well as subsequent tumor volume change, has been observed, suggesting that malignant tumors tend to be more sensitive to CK treatment.

Although clinical trials of small molecule probes conducted worldwide is limited, its potential for early assessment of radiotherapy has been proved to be effective and reliable, as shown in this study and previous preclinical and clinical studies [14, 15, 24]. The study is limited to the small samples size, and further studies are needed with large sample clinical data.

## 5. Conclusions

This study shows [18F]ML-10 PET/CT apoptosis imaging to be a safe and effective clinical method for the assessment of early response of radiotherapy. In [18F]ML-10 PET/CT apoptosis imaging, the tracer uptake in normal brain tissue is lower than that in tumor tissue, thus the anatomic positioning of tumor tissue and surrounding edema area could be accurately identified and visualized. More importantly, [18F]ML-10 PET/CT apoptosis imaging can be used for early prediction of the effectiveness of CK radiotherapy. A significant correlation between the rate of change in [18F]ML-10 uptake in the tumor and the rate of subsequent change in tumor volume was observed. In comparison to the therapeutic response in different cancer types, a rapid response in radioactivity, as well as subsequent tumor volume change, has been observed in malignant tumors, which tends to be more sensitive to CK treatment. Another comparison indicates that the therapeutic response of CK treatment is not significantly correlated with the apoptosis level before CK treatment by the study. Our study has also shown the accuracy of AMIC Ray-Scan 64 PET/CT and safety of CK stereotactic radiosurgery treatment of intracranial tumors in PLAGH.
