# Erratum to “[18F]ML-10 Imaging for Assessment of Apoptosis Response of Intracranial Tumor Early after Radiosurgery by PET/CT”

**DOI:** 10.1155/2019/4967404

**Published:** 2019-03-19

**Authors:** Lu Sun, Kedi Zhou, Weijun Wang, Xiaojun Zhang, Zhongjian Ju, Baolin Qu, Zhizhong Zhang, Jinyuan Wang, Zhipei Ling, Xinguang Yu, Jinming Zhang, Longsheng Pan

**Affiliations:** ^1^Department of Neurosurgery, PLA General Hospital, 28 Fuxing Rd., Haidian District, Beijing, China; ^2^Department of Biomedical Engineering, College of Engineering, Peking University, 5 Yiheyuan Rd., Haidian District, Beijing, China; ^3^Department of Nuclear Medicine, PLA General Hospital, 28 Fuxing Rd., Haidian District, Beijing, China; ^4^Department of Radiation Oncology, PLA General Hospital, 28 Fuxing Rd., Haidian District, Beijing, China

In the article titled “[18F]ML-10 Imaging for Assessment of Apoptosis Response of Intracranial Tumor Early after Radiosurgery by PET/CT” [1], there was an error in the title where the word “Radiotherapy” should be corrected to “Radiosurgery.” The corrected title is shown above and updated in place.
